# A GIS Model Predicting Potential Distributions of a Lineage: A Test Case on Hermit Spiders (Nephilidae: *Nephilengys*)

**DOI:** 10.1371/journal.pone.0030047

**Published:** 2012-01-06

**Authors:** Magdalena Năpăruş, Matjaž Kuntner

**Affiliations:** 1 Institute of Biology, Scientific Research Centre, Slovenian Academy of Sciences and Arts, Ljubljana, Slovenia; 2 Tular Cave Laboratory, Kranj, Slovenia; 3 National Museum of Natural History, Smithsonian Institution, Washington, D.C., United States of America; 4 College of Life Sciences, Hubei University, Wuhan, China; University of Western Ontario, Canada

## Abstract

**Background:**

Although numerous studies model species distributions, these models are almost exclusively on single species, while studies of evolutionary lineages are preferred as they by definition study closely related species with shared history and ecology. Hermit spiders, genus *Nephilengys,* represent an ecologically important but relatively species-poor lineage with a globally allopatric distribution. Here, we model *Nephilengys* global habitat suitability based on known localities and four ecological parameters.

**Methodology/Principal Findings:**

We geo-referenced 751 localities for the four most studied *Nephilengys* species: *N. cruentata* (Africa, New World), *N. livida* (Madagascar), *N. malabarensis* (S-SE Asia), and *N. papuana* (Australasia). For each locality we overlaid four ecological parameters: elevation, annual mean temperature, annual mean precipitation, and land cover. We used linear backward regression within ArcGIS to select two best fit parameters per species model, and ModelBuilder to map areas of high, moderate and low habitat suitability for each species within its directional distribution. For *Nephilengys cruentata* suitable habitats are mid elevation tropics within Africa (natural range), a large part of Brazil and the Guianas (area of synanthropic spread), and even North Africa, Mediterranean, and Arabia. *Nephilengys livida* is confined to its known range with suitable habitats being mid-elevation natural and cultivated lands. *Nephilengys malabarensis*, however, ranges across the Equator throughout Asia where the model predicts many areas of high ecological suitability in the wet tropics. Its directional distribution suggests the species may potentially spread eastwards to New Guinea where the suitable areas of *N. malabarensis* largely surpass those of the native *N. papuana*, a species that prefers dry forests of Australian (sub)tropics.

**Conclusions:**

Our model is a customizable GIS tool intended to predict current and future potential distributions of globally distributed terrestrial lineages. Its predictive potential may be tested in foreseeing species distribution shifts due to habitat destruction and global climate change.

## Introduction

Imagine a map of the world with detailed localities for past, present and future occurrence of species. A tool such as this, if available for multiple species representing most lineages of living beings, could be utilized beyond simple biodiversity assessments. It could serve conservation purposes, landscape planning, tourism, biomedicine, global change monitoring and management of invasive species, to name but a few of numerous potential uses. Such a tool unfortunately does not exist, but the ubiquity of detailed taxonomic revisions and the wide availability, and sophistication, of GIS and machine learning softwares, should make it possible in the very near future. Today, numerous GIS distribution studies exist at local [1]–[5] to continent and global scales using single species [6]. However, to our knowledge, no published study has focused on a global prediction of habitat suitability for a whole evolutionary lineage of species, based on actual specimen records and on ecological simulation [7]. Taxa that are each other's closest relatives have comparable evolutionary ages, comparable life histories as modified from the traits inherited from the common ancestor, and importantly, these taxa have likely experienced similar ecological histories. We argue that studying several closely related species in a spatial and ecological context can offer insights into broader patterns of species ecology and exclusivity than studies confined to single, or several unrelated species. Here, we present such a study on a clade of terrestrial invertebrates—spiders.

Spiders are megadiverse, ubiquitous, and as general predators, they are crucial elements of terrestrial ecosystems [8]. A large percentage of spider diversity belongs to orb weaving spiders, Orbiculariae [9], and these are particularly suitable for global level studies because of their conspicuousness in most biomes. We chose to model global habitat suitability of the orb weaving hermit spiders, genus *Nephilengys*, for several reasons beyond their global reach. First, the genus is taxonomically revised [10], [11], yet with six species remains relatively species poor and thus manageable for such a project. Second, these species range from small island endemics (*N. dodo, N. borbonica*) to species widely spread over continents [10], [11]. Third, this taxonomic and geographic knowledge is based on a wealth of examined specimen records, available as geographic data points [10], [11]. Fourth, there is strong evidence that the six species currently recognized are globally fully allopatric [10], [11], which hints at their geographic, ecological and behavioral exclusivity. Finally, these extremely sexually dimorphic nephilid spiders are becoming model organisms in a range of disciplines [11]–[14] and thus predicting their habitat suitability, or even future occurrence would facilitate further research of their biology.

**Figure 1:**
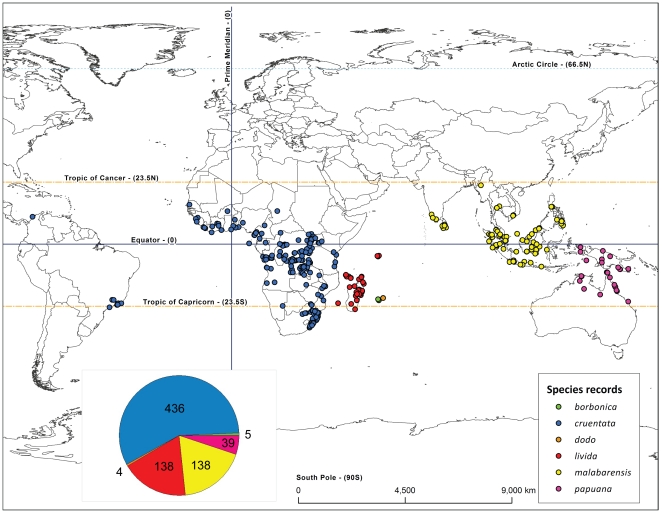
Global distribution of hermit spiders (*Nephilengys* species) based on all available data (Table S1) with the numbers of specimen records per species (inset).

Figure 1 shows the currently known ranges for all six *Nephilengys* species, which are i) tropical, and ii) show a range in inhabited areas from limited island distributions, such as in *N. livida* (Vinson, 1863), *N. borbonica* (Vinson, 1863) and *N. dodo* Kuntner & Agnarsson, 2011, to a wider distribution over several islands as in *N. papuana* Thorell, 1881, and to extremely wide distributions over vast areas as seen in *N. malabarensis* (Walckenaer, 1841) and *N. cruentata* (Fabricius, 1775), the latter being even spread intercontinentally. There is circumstantial evidence, partly from their natural history and partly genetics, that these spiders are moderately good dispersers [12], and as such they probably can travel by air (balloon) long distances as juveniles. However, their establishment in new areas and consequently their fine scale distribution depends on the proximity, accessibility, and, primarily, the ecological suitability of the available space. Their natural history suggests that they need hard vertical surfaces (trees) to anchor their large webs [10], [14]. As any terrestrial organism, they further need spaces of suitable elevation, year round temperature and sufficient precipitation. We thus accounted for these ecological parameters and combined them with all *Nephilengys* specimen records, which we databased and georeferenced. We then devised a GIS model [15] to predict habitat suitability of *Nephilengys* species globally. We discuss the results in the light of potential uses of the model in the future, in particular how the maps may predict species propensity to invade previously uninhabited territories.

## Methods

### Locality data

We used the available *Nephilengys* locality data from a comprehensive taxonomic revision [10] and updated them for taxonomic changes affecting the island species in the Indian Ocean [11]. All georeferenced data contain historic collections from 27 museums [10] and additional specimen records from our recent collecting trips to South Africa (2006), Brazil (2007), the islands of the Indian Ocean (2008, 2010), and Southeast Asia (2007–2011). We used a variety of online gazetteers and Google Earth to georeference the unknown localities, but excluded from this analysis all ambiguous localities. The final list contains 760 specimen records for all known six *Nephilengys* species (see Table S1) and these records are shown on the World map (Fig. 1). However, two species from the Indian Ocean islands of Réunion (*N. borbonica*) and Mauritius (*N. dodo*) are only known from a few localities (Fig. 1; [11]). Our preliminary analysis of all species revealed that these two species are too poorly represented in our georeferenced sample to be meaningful in predicting their ranges. Thus, they were omitted from the model, where we only treat the four species represented with putatively adequate data: *N. cruentata* (specimen records N = 436), *N. livida* (N = 138), *N. malabarensis* (N = 138) and *N. papuana* (N = 39).

### Ecological parameters

Our model (Text S2) considers four ecological attributes for each georeferenced locality that are deemed the most decisive in local spider distribution [8], [15], [16]: elevation (m), annual mean temperature (degrees C), annual mean precipitation (mm), and land cover (Table 1, Table S1). We obtained three ESRI grid layers available on the WORLDCLIM database [17] for the first three parameters, and used the layer with global land cover from the years 1999–2000 [18]. All overlaying grid layers have a spatial resolution of 1 km at the Equator and use WGS84 datum. As global projected coordinate system, we used Cylindrical Equal Area, in order to measure areas without any distortion [19].

**Table 1:** Environmental parameters used for the habitat suitability model.

Ecological parameters	Range and Units^1^	Code
Elevation	0 – 3350 m	ALT
Annual Mean Temperature	−19,4–32°C	TMA
Annual Mean Precipitation	0–11401 mm	PMA
Global Land Cover	Codes from 1 to 23^2^	GLC

1 Range of values included in the maximum search area computed with Directional Distribution with SD3

2 Land-cover legend: 1— Tree cover, broadleaved, evergreen; 2— Tree cover, broadleaved, deciduous, closed; 3 — Tree cover, broadleaved, deciduous, open; 7 — Tree cover, regularly flooded, fresh water; 9 — Mosaic: Tree cover/Other natural vegetation; 11 — Shrub cover, closed-open, evergreen; 12 — Shrub cover, closed-open, deciduous; 13 — Herbaceous cover, closed-open; 14 — Sparse herbaceous or sparse shrub cover; 15 — Regularly flooded shrub and/or herbaceous cover; 16 — Cultivated and managed areas; 17 — Mosaic: Cropland/Tree cover/Other natural vegetation; 20 — Water bodies; 22 — Artificial surfaces and associated areas.

### Statistical Analyses

Our model is based on regression analysis using all four ecological parameters (TMA, PMA, ALT and GLC; Table 1) as independent variables [20], [21], with each geographical coordinate per species, and hence habitat suitability, representing a dependent variable [22], [23]. This results in a probability distribution of the ecological conditions at each location [24]. To test which of the four parameters have the highest influence on species distribution, and to select two best fit parameters, we performed a linear backward regression analysis using spatial statistics in ArcGIS 9.3.1 [25], which employs Ordinary Least Squares (OLS) and Geographically Weighted Regression (GWR) to compute linear regression. OLS is the more general analysis representing a global modeling regression using all four ecological parameters. The OLS analysis assesses model performance through various results including the robust probability, the Variance Inflation Factor (VIF) and the two metrics reported in Table 2, namely the adjusted R^2^ and the Akaike Information Criterion (AIC; [26]). The OLS regression helps us to detect multicollinearity severity through VIF, an index measuring how much the variance of the estimated regression coefficient was increased because of collinearity. Explanatory variables with a VIF greater than 7.5 were removed one by one, until the obtained model became unbiased. Also, the robust probability has indicated the most statistically significant variables. After examining the robust probability and VIF values, we re-ran OLS removing the variables following backward linear regression until the remaining variables stabilized.

**Table 2:** OLS and GWR regression results yielding two best parameters for each species habitat suitability model (see Methods for details).

Species ecological parameters	OLS results	GWR results	Spatial Autocorrelation (Global Moran's I)	
*N. cruentata*	AIC: 5380.4	Neighbours: 10	Moran's Index:	0.061917
TMA, ALT	R^2^Adjusted: 0.17	AICc: 228.9	Expected Index:	−0.011111
		R^2^: 0.92	Variance:	0.018019
		R^2^Adjusted: 0.81	Z Score:	0.544034
			p-value:	0.586418
			Random	
*N. livida*	AIC: 1403.1	Neighbours: 10	Moran's Index:	−0.349273
ALT, GLC	R^2^Adjusted: 0.12	AICc: 279.5	Expected Index:	−0.020833
		R^2^: 0.96	Variance:	0.034546
		R^2^Adjusted: 0.92	Z Score:	−1.767073
			p-value:	0.077216
			Quasi-random	
*N. malabarensis*	AIC: 1414.7	Neighbours: 10	Moran's Index:	−0.260731
PMA, GLC	R^2^Adjusted: 0.10	AICc: 526.0	Expected Index:	−0.008333
		R^2^: 0.84	Variance:	0.006664
		R^2^Adjusted: 0.67	Z Score:	−3.091786
			p-value:	0.001990
			Quasi-dispersed	
*N. papuana*	AIC: 300.3	Neighbours: 10	Moran's Index:	0.132592
PMA, GLC	R^2^Adjusted: 0.12	AICc: 158.0	Expected Index:	−0.038462
		R^2^: 0.77	Variance:	0.097500
		R^2^Adjusted: 0.52	Z Score:	0.547811
			p-value:	0.583822
			Random	

GWR then uses the two best ecological parameters from the OLS to model the dependent variable (habitat suitability) for each species. We selected the following settings in GWR [27]: the number of nearest neighbors = 10; Bandwidth Method = Bandwidth Parameter; Kernel Type = Adaptive. As above, we evaluated the GWR results by the computed AIC and adjusted R^2^ values (Table 2). GWR also visualizes the results, as it provides coefficient surface maps for each ecological parameter thereby helping detect where the strongest relationships are [28].

Finally, we ran spatial autocorrelation (Global Moran's I) to estimate whether in the final combination of ecological parameters the residuals exhibit a random spatial pattern. The autocorrelation calculates the Moran's I Index value and both p-value and Z score, evaluating the significance of the index [29]. The null hypothesis states that there is no spatial clustering of the values associated with the geographic features in the study area. When the p-value is small and the absolute value of the Z score is large enough that it falls outside of the desired confidence level, the null hypothesis can be rejected. If the Moran index value is greater than 0, the set of features exhibits a clustered pattern. If the value is less than 0, the set of features exhibits a dispersed pattern. In a good model the residuals reflect random noise [28].

### The Model

**Figure 2:**
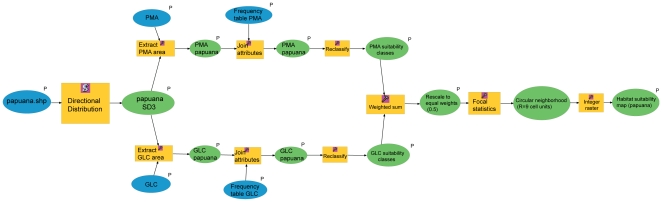
Diagram showing the model created in ModelBuilder: the case of *Nephilengys papuana*, shown here, where two best fit parameters, land cover (GLC) and precipitation (PMA) were used to predict habitat suitability.

The main concept of our model (Text S2) is to build an area within each species potential reach (see directional distribution below) and to search within this area the points with specific values for the selected two ecological parameters that are more or less likely to represent suitable species habitat. We created our GIS model using ModelBuilder, a graphic programming environment within ArcGIS Desktop 9.3.1 [25], which visualizes the work flow (Fig. 2) with all chained data and processes [30]. The model combines vector data (specimen records as point layers), raster data (ESRI grids for ecological parameters at the global scale) and stand-alone tables, in order to summarize and store properly specific information [7], [31].

**Figure 3:**
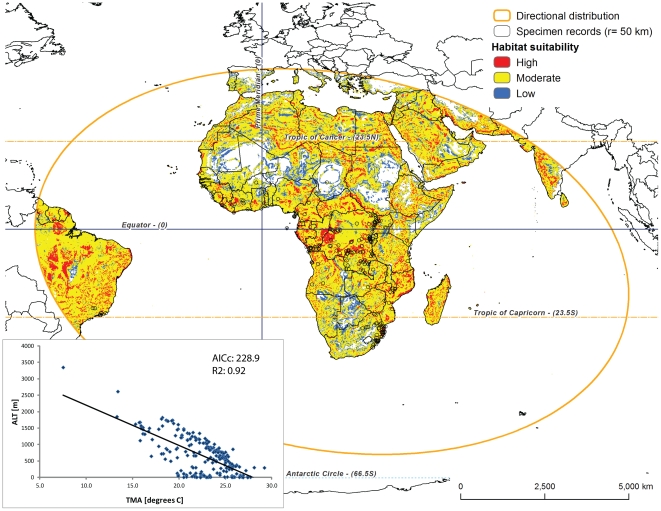
Predicted habitat suitability for *Nephilengys cruentata* within its directional distribution area (see Methods for details). The model builds on two best fit parameters, temperature (TMA) and altitude (ALT, see inset with GWR results from Table 2). Probability dots have an area of 235.7 km^2^, and the specimen record circles are 7853.8 km^2^.

**Figure 4:**
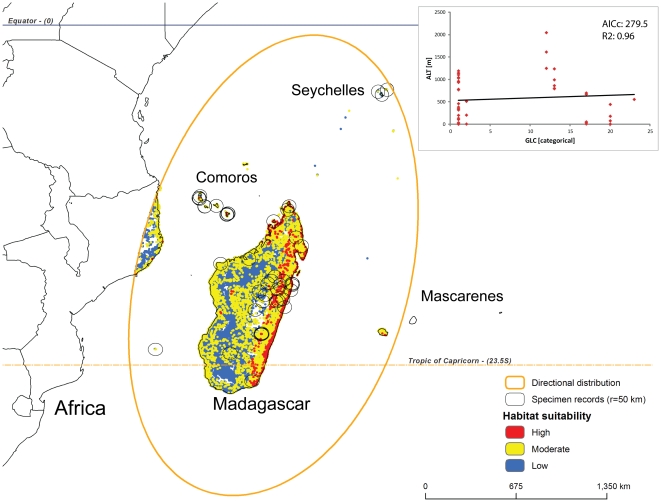
Predicted habitat suitability for *Nephilengys livida* within its directional distribution area (see Fig. 3 and Methods for details). The model builds on two best fit parameters, land cover (GLC) and altitude (ALT, see inset with GWR results).

**Figure 5:**
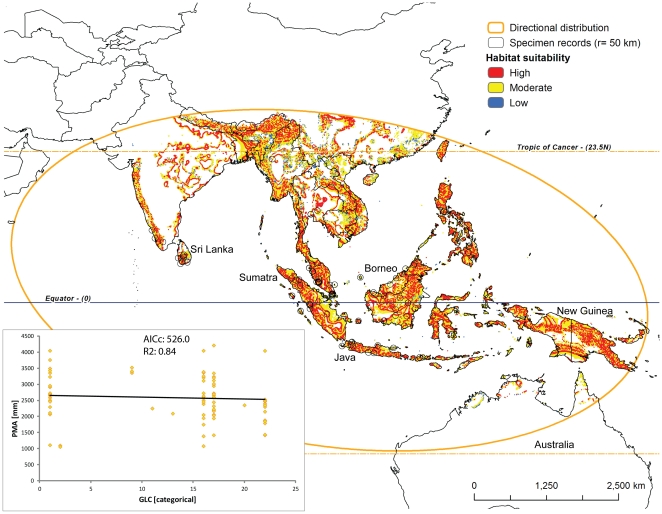
Predicted habitat suitability for *Nephilengys malabarensis* within its directional distribution area (see Fig. 3 and Methods for details). The model builds on two best fit parameters, land cover (GLC) and precipitation (PMA, see inset with GWR results).

**Figure 6:**
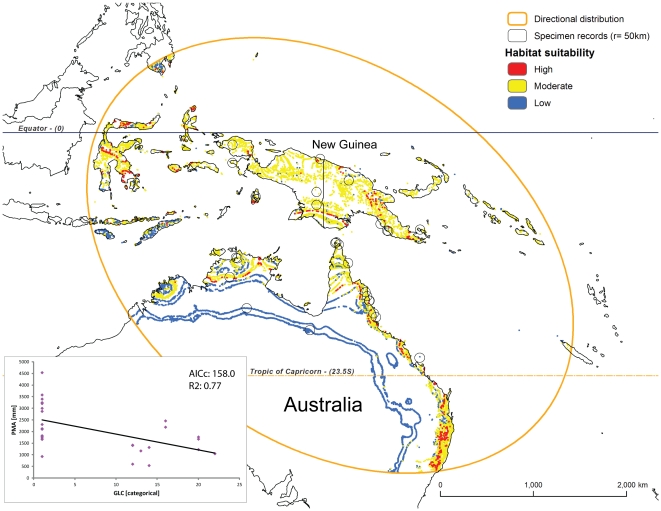
Predicted habitat suitability for *Nephilengys papuana* within its directional distribution area (see Fig. 3 and Methods for details). The model builds on two best fit parameters, land cover (GLC) and precipitation (PMA, see inset with GWR results).

We computed the *directional distribution* (shown as orange ellipse in Figs. 3, 4, 5, and 6) for each species as elliptical polygon centered on the mean of all localities. Among the options of the ellipse extent (one, two, or three standard deviations), we chose the latter in order to maximize the potential species distribution to cover approximately 99% of all feature centroids [28]. This ellipse summarizes the central tendency and the spatial orientation of existing specimen records, which is informative of potential species dispersion. We used all terrestrial habitats within this ellipse as each species potential target area. Biologically, such areas of interest implicitly assume that they are within each species dispersal range, and that they are not inhabited by competing species. Especially the latter assumption is oversimplified as is clear from Figure 1. However, such assumption is not unwarranted for this model, as *Nephilengys* species in fact show a complete species separation. In other words, their global distribution is fully allopatric (Fig. 1, [10]).

We extracted the values of the ecological parameters in stand-alone tables by running a simple script (Text S1), and obtained their observed frequency, then logically grouped them into three classes: high, moderate and low frequency (Table S2). Within each species directional distribution we then identified all values corresponding to these classes for the two best correlated ecological parameters (Table 2). We renamed these classes to now represent a species *habitat suitability* (high = 3, moderate = 2, low = 1), while all outlying values were ignored. The resulting two ecological maps thus only contained the cells ranked as 3, 2 or 1 for high/moderate/low predicted habitat suitability for a species. Both maps were then combined by receiving equal weight. We finalized our prediction analysis by adjusting the habitat suitability dot size to fit the global scale of our analysis; using Focal statistics [15] the circular area was changed from the default radius value of 3 to 9 cell units, which corresponds to a radius of 8.67 km. All cells whose center falls inside this radius were included in processing the neighborhood. The resulting habitat suitability is visualized on species maps (Figs. 3, 4, 5, and 6). Although the scales of these maps vary, all circles representing actual specimen records are equal sized (r = 50 km; 7853.83 km^2^), as are the colored habitat dots representing three suitability classes (235.73 km^2^).

## Results

Based on the outcomes of the OLS and GWR regressions (Table 2), we selected the following ecological parameter pairs for species models: TMA and ALT for the *N. cruentata* model (Fig. 3 inset), GLC and ALT for the *N. livida* model (Fig. 4 inset), and GLC and PMA for both *N. malabarensis* and *N. papuana* models (Figs. 5 and 6 inset). The species also differ in the type of relationships between these variables. The *N. cruentata* OLS model shows a strong positive correlation with temperature (OLS: β = 19.8; p<0.0001) meaning high habitat suitability in warmer areas (Table S2), and a positive correlation with altitude (OLS: β = 0.12; p<0.0001) meaning high habitat suitability at the elevation around 550 m (Table S2). The *N. livida* OLS model shows a slightly negative correlation with altitude (OLS: β = −0.02; p = 0.0004) meaning high habitat suitability at elevations 500 to 700 m (Table S2, Fig. 4 inset), and a positive correlation with land cover (OLS: β = 1.09; p = 0.04) meaning preferences for a mix of natural vegetation and cultivated areas (Table S2). The *N. malabarensis* OLS model, first ambiguous but settling after several runs, shows a slightly positive correlation with precipitation (OLS: β = 0.001; p = 0.6) meaning high habitat suitability in the wet tropics over 2000 mm (Table S2, Fig. 5 inset), and a slightly positive correlation with land cover (OLS: β = 0.05; p = 0.9) meaning preferences for a mix of natural vegetation and cultivated areas (Table S2). In contrast, the *N. papuana* OLS model shows a negative correlation with precipitation (OLS: β = −0.77; p = 0.3) meaning high habitat suitability in the dry tropics averaging 1500 mm (Table S2, Fig. 6 inset), and a negative correlation with land cover (OLS: β = −0.77; p = 0.02) meaning preferences for natural vegetation (Table S2). The results of the spatial autocorrelation analysis, the *N. cruentata* and *N. papuana* models indicated random residuals, while *N. livida* had quasi-random residuals and *N. malabarensis* had quasi-dispersed residuals (Table 2).

The four species, for which we provide spatial analyses, differ in the total area they cover as well as in the ecological parameters of their known localities (Tables S1 and S2). *Nephilengys cruentata* is currently known from most regions within the tropical and subtropical Africa and from an Atlantic coastal region of Brazil and Colombia (Figs. 1, 3), where it is most likely introduced [10]. However, our model predicts that all over Africa there exist more or less continuous areas with the species' inferred moderate and high habitat suitability, and only in the most arid parts of Africa the habitats are unsuitable (Fig. 3). The species habitat suitability also extends further out of Africa into the Mediterranean and into Arabia and Asia and onto Madagascar, all of which are areas currently not harboring *N. cruentata*. Moreover, in South America there is a vast expanse of areas between Brazil and the Guiana shield where the species, if indeed non-native, might very likely spread in the future due to habitat suitability (Fig. 3, note the absence of any currently known records there). The directional distribution of *N. cruentata*, denoted as ellipse in Figure 3, largely surpasses the actual distribution of the species, reaching into Europe in the north and Asia as well as a large expanse of the Indian Ocean in the east.

*Nephilengys livida* is currently known from Madagascar and adjacent islands in the northwestern part of the Indian Ocean [11] (Figures 1, 4). Based on the existing records, our model identifies areas, in particular in eastern Madagascar, where their local distribution is more likely than in the southern and western arid zones (Fig. 4). Its directional distribution fits the known species range well as the ellipse includes the Comoro island chain and that of Seychelles and only slightly touches the coast of East Africa, which is already outside of the species range (Fig. 4). *Nephilengys malabarensis* is currently known from the tropical and subtropical parts of South and Southeast Asia [10], where the model predicts many areas of high and moderate habitat suitability for this species (Fig. 5). The species directional distribution is longitudinally spread over the Equator, however, it largely surpasses the species eastward range into areas falling outside of the species range such as New Guinea (Fig. 5). The known range of *N. papuana* is between western New Guinea into Australia, but with an apparent absence from all neighboring minor islands [10], while the model predicts its directional distribution to reach much further northwest and southeast into the tropical Southeast Asia and subtropical Australia. There, an interesting pattern shows narrow strips of coastal areas with mostly moderate habitat suitability, and a narrow belt of low suitability bordering the arid expanse in Australia (Fig. 6).

## Discussion

Various ecological modeling approaches exist that quantify the relationships between the species and their environment with the goal of predicting species distributions or habitat suitability (e.g. Maxent [22], [23], GLM [32], DIVA GIS [33]). Specialized software packages have been used for this purpose, and there is no clear justification for using one over the other (e.g. Maxent versus BIOCLIM [34], Maxent versus DIVA GIS [35], BIOCLIM, DOMAIN, GARP and Maxent comparison [36]). Our approach is not to follow a traditional niche modeling or a species distribution modeling, but rather to offer a GIS based model to easily identify most suitable areas for a group of species based on particular environmental factors. Our model is easy to run, has a high visual content, and uses simple and widespread GIS tools, which can be implemented as a chain of processes in a single operation. Additionally, the script can be customized and used for organisms other than our target group.

To our knowledge, our study is the first of this kind to model habitat suitability, which may be indicative of current and future species distributions, of an entire genus whose species exhibit a fully allopatric and global distribution. The data which we intended to model reach 760 specimen records (Table S1), but these range in species coverage from only a handful and too few to model (*N. borbonica, N. dodo*) through moderate (*N. papuana*) to well represented (*N. malabarensis, N. livida*) and even extremely well sampled (*N. cruentata*). We believe the final models (Figs. 3, 4, 5, and 6) performance is adequate for the intended purpose, although the spatial autocorrelation results suggest that the *N. cruentata* and *N. papuana* models are best due to random residuals, while *N. livida* and *N. malabarensis* are not fully random (Table 2). Compared with OLS regression analyses, the GWR regression produced much better results in all species models with lower AIC and higher R^2^ values (Table 2). The GWR R^2^ values, measuring the goodness of fit for the selected ecological parameter pairs, showed values over 75% in all models. They differed in performance, which we believe relates to the number of data points available for each species and their dispersion. In addition to a R^2^ over 90%, the desired feature of a reliable model is a small difference between R^2^ and the adjusted R^2^ (maximum 0.15 [28]). Even the model containing the least data (39 for *N. papuana*) showed a satisfactory correlation between the two parameters at R^2^ = 77%. The two models containing 138 data points each (*N. malabarensis, N. livida*) had better to best performance (R^2^ = 84%; R^2^ = 96%), while the data richest model (436 records for *N. cruentata*) also performed very well (R^2^ = 92%).

The directional distribution, usually forming an ellipse (Figs. 3, 4, 5, and 6), uses the geostatistical mean center of the factual species localities, then takes three SDs (approximately 99%) of all georeferenced localities to predict the species total, or potential range, which reflects the spatial orientation of existing specimen records [28]. Although geostatistically clearly defined, the biological significance of the directional distribution remains obscure. To our knowledge, the closest use of the directional distribution to biology has been in modeling the spread of diseases [37], [38]. Our interpretation of its significance is that this shape defines a potential range to where individuals of the species in question may reasonably reach via dispersal. The directional distribution, however, ignores the ranges of other existing, neighboring species, and thus ignores interspecific competition for space and resources. Likewise, it fails to account for human mediated colonization of new areas, which may flaw the interpretations of synanthropic species, e.g. *N. cruentata*. Nevertheless, we discuss the calculated shapes of *Nephilengys* directional distributions along the lines of potential range of natural dispersal, although interpretations could differ in the case of other organisms with overlapping species ranges.

The directional distribution of *N. cruentata* (Fig. 3) fails to account for the lone Colombian coastal record (compare with Fig. 1), which is clearly due to human assisted dispersal. However, it reaches certain regions outside of the species actual range, such as Madagascar, India and Sri Lanka, all occupied by other *Nephilengys* species (Fig. 1) but also showing as suitable for *N. cruentata* (Fig. 3). Interestingly, the model also predicts a large part of Northern Africa and the Mediterranean as suitable for this species despite the fact that no *Nephilengys* has ever been recorded there, and despite the actual species range not reaching as far north as the Sahara. In the case of the *N. cruentata* northern limits and predictions, our model, which predicts *N. cruentata* habitat suitability in warm tropical areas of medium altitude, could be used in the future to test potential species distribution shifts due to climate change [39]. Similarly, the Western hemisphere part of the model clearly and boldly predicts potential avenues for the species invading large tracts of South America. Naturally, the future limits of the *N. cruentata* directional distribution will shift depending on the species spreading into currently unpopulated territories. Another area of interest considering species spreading and potential invasiveness, perhaps linked to humans, is the Mozambique Channel off east Africa. Due to its smaller size compared to the Atlantic Ocean, the likely wind assisted dispersal in nephilid spiders [12] and the relative proximity of related species in the islands off Africa's coast [11], we find it likely that these islands may stage occasional cases of interspecific spreading and direct competition. Our *N. cruentata* model indeed predicts habitat suitability for this species over most of Madagascar, Comoros and Mascarenes. A recent biogeographic study found phylogenetic evidence of colonization of *Nephilengys* populations from Africa onto Madagascar and further east, each such spread followed by new species evolution [11]. Our spatial model clearly supports such scenario.

In contrast to *N. cruentata*, the directional distribution of *N. livida*, denoted as ellipse in Fig. 4, in fact mirrors its real distribution well, as it only touches the coast of east Africa and the island of Réunion, both regions falling just outside of the species range. The model here predicts the species habitat suitability based on the combination of mid elevation and mixed natural and altered land cover. Although this suggests that *N. livida* inhabits both forests and cultivated areas, this species is less synanthropic than *N. cruentata* [11], and thus its invasiveness is clearly limited compared with *N. cruentata*. As noted above, Kuntner and Agnarsson [11] found evidence for a Madagascar origin of the Mascarene *Nephilengys* fauna, and again, a low probability of *N. livida* occurring on Réunion, where another species, *N. borbonica* is native, supports such colonization pattern. Another contrast to *N. cruentata* is the north-south shape of *N. livida* directional distribution (Fig. 4), but this may reflect the shape and direction of the available territory, notably Madagascar.

As in *N. cruentata*, the directional distribution of the *N. malabarensis* model is east-west, clearly centered around the Equator (Fig. 5). A further resemblance between the two models is the vast geographical area covered by the species, in *N. malabarensis* nearly entire tropical Asia. We believe these facts reflect both species synanthropic habits. *Nephilengys malabarensis* is common in coastal and higher elevation areas over SE Asia, where its habitat ranges from native forest to introduced tree stands to houses [14], which is also accurately reflected by our model predicting high habitat suitability in wet areas of the mixed land cover types. Despite its apparent absence from the areas that biogeographically represent Australasia (e.g. Moluccas, New Guinea; [10]), our model predicts a vast potential range with many pockets of highly suitable habitat there. As a curiosity, the name for the species refers to the coast of Malabar (W India). Although we lack actual locality data north from Kerala, our model predicts habitat suitability for *N. malabarensis* over the entire western Indian coastal area.

The *N. papuana* directional distribution seems to mirror its known distribution NW-SE fairly accurately (Fig. 6). The species habitat suitability is defined in the model by the combination of the same two parameters as in *N. malabarensis.* However, the preferences are quite opposite hinting at both geographical and ecological exclusivity of the two species. Highest *N. papuana* habitat suitability is namely in the drier (sub) tropical native forests.

### Conclusions

*Nephilengys cruentata* suitable habitats span most of Africa, where it has traditionally been known as a prominent and ecologically important invertebrate, a large part of Brazil and into the Guiana shield, where it has spread synanthropically in the past centuries and is predicted to continue this trend, and even Northern Africa and the Mediterranean, where it has never been recorded. Although annual mean temperature is suitable there, we nevertheless believe the winter extremes might be too harsh to support any viable populations. However, it would be interesting to predict the species potential reach in the light of global climate change in the Mediterranean, and due to potential invasiveness in South America. *Nephilengys livida* and *N. papuana* are confined to their known ranges and they seem to occupy mixed (*N. livida*) and natural forested areas (*N. papuana*), and thus show fewer propensities to spread. *Nephilengys malabarensis*, however, ranges across the Equator throughout Asia with many areas of high ecological suitability, and the model predicts that the species may potentially spread further eastwards, as e.g. New Guinea contains many more areas suitable for the non-native *N. malabarensis* (Fig. 5) compared to the native *N. papuana* (Fig. 6).

Vink and colleagues [39] modeled a potentially global distribution of the invasive widow spider, *Latrodectus hasselti*, which had invaded New Zealand and Japan from Australia, and may represent a global health hazard due to its venom. Similar to a single species model but more inclusive, our *Nephilengys* model represents a tool to predict current and future habitat suitability of globally distributed, fully allopatric, and at least in part, synanthropic and invasive, clade of prominent invertebrates. This tool will easily be tested in the future, especially evaluating the effects that global climate change and habitat destruction may play in invertebrate ecology. It is our hope that our model will be applied to other organisms with similarly global distributions, regardless of their biology.

## Supporting Information

Table S1 **A list of georeferenced localities used in the four species model of** ***Nephilengys*** **global habitat suitability, with their corresponding attributes (annual mean temperature, annual mean precipitation, land cover and altitude).**
(XLS)

Table S2 **Frequencies and frequency classes (high, moderate, low) for all four ecological parameters used to predict** ***Nephilengys*** **global habitat suitability.**
(XLS)

Text S1 **The script for generating frequency classes (high, moderate, low) for the four ecological parameters used to predict** ***Nephilengys*** **global habitat suitability.** A Python script file (py) is available at ezlab.zrc-sazu.si.
(TXT)

Text S2 **The model script predicting** ***Nephilengys*** **global habitat suitability.** A Python script file (py) is available at ezlab.zrc-sazu.si.
(TXT)
